# Early inflammatory changes in radiation-induced oral mucositis: Effect of pentoxifylline in a mouse model

**DOI:** 10.1007/s00066-017-1119-8

**Published:** 2017-03-03

**Authors:** Sylvia Gruber, Eva Bozsaky, Eva Roitinger, Karoline Schwarz, Margret Schmidt, Wolfgang Dörr

**Affiliations:** 1 Applied and Translational Radiobiology, Dept. Radiation Oncology/CD Lab. Med. Radiation Research for Radiation Oncology, Medical University/AKH Vienna, Waehringer Guertel 18–20, 1090 Vienna, Austria; 2 Dept. Radiation Oncology, Faculty of Medicine and University Hospital Carl Gustav Carus, Technische Universität Dresden, Fetscherstr. 74, 01307 Dresden, Germany; 3 OncoRay – National Center for Radiation Research in Oncology, Faculty of Medicine and University Hospital Carl Gustav Carus, Technische Universität Dresden, Helmholtz-Zentrum Dresden – Rossendorf, Fetscherstr. 74, 01307 Dresden, Germany

**Keywords:** Fractionated irradiation, Oral mucositis, Head and neck cancer, Radiation protection, Animal model, Fraktionierte Bestrahlung, Orale Mukositis, Kopf-und-Hals-Tumore, Strahlenschutz, Tiermodell

## Abstract

**Purpose:**

Early inflammation is a major factor of mucosal reactions to radiotherapy. Pentoxifylline administration resulted in a significant amelioration of radiation-induced oral mucositis in the mouse tongue model. The underlying mechanisms may be related to the immunomodulatory properties of the drug. The present study hence focuses on the manifestation of early inflammatory changes in mouse tongue during daily fractionated irradiation and their potential modulation by pentoxifylline.

**Materials and methods:**

Daily fractionated irradiation with 5 fractions of 3 Gy/week (days 0–4, 7–11) was given to the snouts of mice. Groups of 3 animals per day were euthanized every second day between day 0 and 14. Pentoxifylline (15 mg/kg, s. c.) was administered daily from day 5 to the day before sacrifice. The expression of the inflammatory proteins TNFα, NF-κB, and IL-1β were analysed.

**Results:**

Fractionated irradiation increased the expression of all inflammatory markers. Pentoxifylline significantly reduced the expression of TNFα and IL-1β, but not NF-κB.

**Conclusion:**

Early inflammation, as indicated by the expression of the inflammatory markers TNFα, NF-κB, and IL-1β, is an essential component of early radiogenic oral mucositis. Pentoxifylline differentially modulated the expression of different inflammatory markers. The mucoprotective effect of pentoxifylline does not appear to be based on modulation of NF-κB-associated inflammation.

## Zusammenfassung

**Hintergrund und Ziel:**

Frühe entzündliche Veränderungen sind ein bedeutender Faktor während der Strahlenreaktion der Schleimhaut. Die Behandlung mit Pentoxifyllin erzielte eine signifikante Minderung strahleninduzierter oraler Mukositis im Mauszungenmodel. Die zugrundeliegenden Mechanismen sind potenziell auf die immunomodulatorischen Eigenschaften des Wirkstoffs zurückzuführen. Die vorliegenden Untersuchungen fokussieren daher auf die Manifestation früher entzündlicher Veränderungen in der Mauszunge während täglich fraktionierter Bestrahlung und deren potenzieller Modifikation durch Pentoxifyllin.

**Material und Methoden:**

Täglich fraktionierte Bestrahlung mit 5 Fraktionen zu 3 Gy/Woche (Tage 0–4 und 7–11) wurde auf die Schnauzen der Mäuse appliziert. Gruppen von je 3 Tieren wurden in 2‑tägigem Abstand eingeschläfert. Pentoxifyllin (15 mg/kg, s. c.) wurde täglich von Tag 5 bis zum Tag vor der Euthanasie verabreicht. Die Expression der Entzündungsproteine TNFα, NF-κB und IL-1β wurde analysiert.

**Ergebnisse:**

Die fraktionierte Bestrahlung erhöhte die Expression aller entzündlicher Marker. Pentoxifyllin reduzierte die Expression von TNFα und IL-1β signifikant, nicht jedoch die Expression von NF-κB.

**Schlussfolgerung:**

Frühe Entzündung, gekennzeichnet durch die Expression der entzündlichen Marker TNFα, NF-κB und IL-1β, ist eine essenzielle Komponente der frühen radiogenen oralen Mukositis. Pentoxifyllin modulierte die Expression jener Marker auf unterschiedliche Weise. Der mukoprotektive Effekt von Pentoxifyllin scheint nicht auf NF-κB-assoziierter antientzündlicher Wirkung zu beruhen.

## Introduction

Oral mucositis is the most important early radiotherapy-related adverse event in head-and-neck cancer patients [1, 2]. Severe confluent epithelial radiation reactions, experienced by the majority of patients, significantly impact the patients’ quality of life with pain, swallowing, and speaking difficulties [3]. Mucositis-related hospitalization and treatment interruptions [4] compromise the therapeutic outcome [5]. Current prophylactic and interventional strategies are purely symptomatic [6]. Oral mucositis is associated with a pronounced early inflammatory response. This offers a target for biology-based mucositis-preventing strategies [7, 8].

Pentoxifylline (PTX), a nonspecific phosphodiesterase inhibitor, may improve tumor oxygenation due to rheologic effects, but may also ameliorate treatment-associated normal tissue morbidity through modulation of inflammatory changes [9]. When tested in a preclinical model of radiation-induced early oral mucositis, PTX was found to significantly reduce the incidence of mucosal ulceration in daily fractionation studies in the established mouse tongue model. PTX treatment yielded the most pronounced radioprotective effect, when the administration interval included the second treatment week of fractionation. This is the time when repopulation, the adaptive epithelial radiation response [10], is already fully active. Time course parameters of oral mucositis, i. e., latent time to the onset of mucosal ulcerations and their respective duration, however, were found virtually unchanged. [11].

The present study was then initiated in the same experimental model in order to characterize inflammation-related biological mechanisms of action of PTX. For this, the expression of the key inflammatory mediators interleukin-1 (IL-1-)β, tumor necrosis factor (TNF)α, and nuclear factor kappa-light-chain-enhancer of activated B cells (NF-κB) during daily fractionated irradiation and the effect of additional PTX treatment was quantified in immunohistochemical investigations. Inflammatory changes are frequently observed during the development of oral mucositis and, hence, present an option to develop a biology-oriented treatment strategy. NF-κB is hypothesized to be one of the key signaling molecules in this aspect [8, 12] and was found up-regulated in biopsies of oral mucosa of patients undergoing myoablative therapy [8]. NF-κB is an evolutionary-conserved signaling molecule that, as an inducible transcription factor, regulates reactions to changes in the environment. Although involved in the transcriptional control of various genes, its main function is the regulation of the immune system. NF-κB is activated upon numerous stimuli, following either a classical, an alternative, or an atypical pathway. Differences in the activation pathways arise from different subsets of stimulatory molecules and, furthermore, from subsequently recruited and differently processed intracellular binding partners. However, all pathways lead to NF-κB activation via inducible degradation of the inhibitory protein complex that sequesters NF-κB in the cytoplasm, followed by NF-κB nuclear translocation and gene transcription [13]. It activates proinflammatory cytokines, chemokines, growth and survival factors [14]. This includes TNFα, which in turn impacts on NF-κB expression in a positive feedback loop and presents the model stimulus for the classical NF-κB activation pathway. TNFα mainly regulates antiapoptotic genes and, aberrantly expressed, is involved in several pathologic conditions, e. g., rheumatoid arthritis [15]. IL-1β presents another NF-κB-activated protein, which can potentiate its own synthesis in autoregulatory pathways and activates NF-κB in return [16, 17]. IL-1β is produced by activated macrophages and is involved in various cellular processes. Most importantly, it is a key mediator of the inflammatory response [18].

## Material and methods

### Animals and housing

In the present experiments, mice of the inbred C3H/Neu strain from the breeding facility of Medical Faculty Carl Gustav Carus, Dresden, Germany were housed under specified pathogen-free conditions with controlled temperature (21–24 °C) and humidity (30–50%). An automated light program provided a 12/12-h light/dark rhythm, with lights on from 06:00 am to 06:00 pm. Maximum ten animals were kept in size 3 Macrolon® cages on saw dust bedding (Sniff ¾, Altrogge, Lage, Germany) with free access to standard mouse diet (Altromin 1326, Altrogge, Lage, Germany) and filtered city tap water from standard perspex drinking bottles.

### Irradiation technique

The technique for irradiation of oral mucosa was described in detail elsewhere [11, 19]. In brief, percutaneous irradiation of the entire snouts of the animals was performed with an YXLON MG325 device (Yxlon International X‑ray GmbH, Hamburg, Germany), operated at 200 kV with a tube current of 20 mA. The animals were guided into plastic tubes (inner diameter 28 mm). Conical holes in a perspex block at the front end of the tubes served for standardized positioning of the snouts. The back ends of the tubes were closed to prevent withdrawal of the animals. Eight animals were irradiated simultaneously. The bodies of the mice were shielded with 6 mm of lead equivalent MCP-96 (HEK Medizintechnik, Lübeck, Germany); the treatment field encompassed the snouts including the entire tongue. The dose homogeneity between the individual snout irradiation fields was ±3%.

### Experimental design

Daily fractionated irradiation with 5 fractions of 3 Gy/week was applied over 2 weeks (days 0–4, 7–11). The study comprised three experimental arms: irradiation alone (IR), irradiation in combination with PTX administration (IR+PTX), and PTX treatment alone (PTX). PTX was administered subcutaneously at a dose of 15 mg/kg from day 5 until the day before sacrifice; on irradiation days, the drug was given 1 h before irradiation in the IR+PTX arm. In both arms, groups of animals (*n* = 3) were sacrificed every second day, and their tongues excised at the base for further investigations. Three untreated and unirradiated mice served as a control group.

### Histological preparation

The tongues were incubated in 4% paraformaldehyde for 24–48 h, cut along the median line, and subjected to routine paraffin embedding. Sections of 3 µm were mounted on Superfrost® plus charged glass slides (Gerhard Menzel GmbH, Braunschweig, Germany) and dried at 37 °C overnight. Subsequently, the sections were deparaffinized and rehydrated through xylene and a graded alcohol series. Heat-mediated antigen retrieval for IL-1β and NF-κB was performed with citrate buffer, pH 6.0, boiling in a microwave set to full power for 20 min. For TNFα, epitope unmasking was performed with EDTA buffer, pH 9.0, for 10 min, also boiling in a microwave set to full power. Endogenous peroxidase activity was blocked (3% hydrogen peroxide, 10 min). The sections were then incubated with normal goat serum (1:200), using a Vectastain® ABC Kit (Vectastain ABC Kit, Vector Laboratories, Burlingame, CA, USA), for 1 h at room temperature, followed by overnight incubation at 4 °C with the primary antibodies. Anti-TNFα (Abcam, Cambridge, MA, USA; Cat. no. 6671; rabbit polyclonal) was used at a concentration of 1:700, anti-IL-1β (Novus Biologicals, Littleton, CO, USA; Cat. no. NBP1-19775; rabbit polyclonal) at a concentration of 1:400 and anti-NF-κB p50 (Abcam, Cambridge, MA, USA; Cat. no. 7971; rabbit polyclonal) at a concentration of 1:100. These concentrations had been defined in previous protocol optimization studies. A second section on the same slide was incubated with the same concentration of rabbit IgG (Dianova GmbH, Hamburg, Germany; Cat. no. 011-000-003) and served as a negative control. The secondary antibody was added for 1 h at room temperature. Subsequently, the sections were incubated with the avidin–biotin complex solution for 1 h at room temperature. The enzyme reaction was visualized by 3,3-diaminobenzidine (DAB) substrate (Vectastain ABC Kit, Vector Laboratories, Burlingame, CA, USA). Nuclear counterstaining was performed with hematoxylin (5 min). Then, the slides were dehydrated in a graded alcohol series, cleared in xylene and coverslipped.

### Histological analysis

Analysis was performed with an Olympus light microscope at 400× magnification. Cytoplasmic TNFα expression could not be attributed to individual cells, hence, not the fraction of expressing, positive cells, but the general staining intensity was determined, separately for the germinal (proliferative) and the functional (postmitotic) nucleated layers of the epithelium. The signal intensity, corresponding to the amount of secreted protein, was assessed semiquantitatively with an arbitrary score from 0 (no signal), 1 (weak signal), 2 (intermediate signal) to 3 (strong signal). The fraction of NF-κB p50 positive cell nuclei was evaluated separately for the germinal and the functional epithelium. In addition, the respective staining intensity was scored as described above for TNFα. IL-1β was not found in the epithelium, but exclusively expressed in macrophages in underlying tongue tissues. IL-1β positive macrophages were counted within at least 15 visual microscopic fields. The number of IL-1β expressing macrophages in unirradiated and untreated control tongue sections was set to 100%, all further day- and experimental arm specific mean values refer to this normalization. The respective staining intensity was determined (see above).

### Statistical analysis

For statistical analysis, the SPSS statistical software (SPSS Inc., Chicago, IL, USA) was used. Mean values and standard deviation (SD) were calculated for each animal, which then served to calculate the mean and standard error for each experimental group. The analysis of variance (one-way ANOVA) was used to test for the significance of a difference between the mean values. A *p*-value of <0.05 was regarded statistically significant.

## Results

**Fig. 1:**
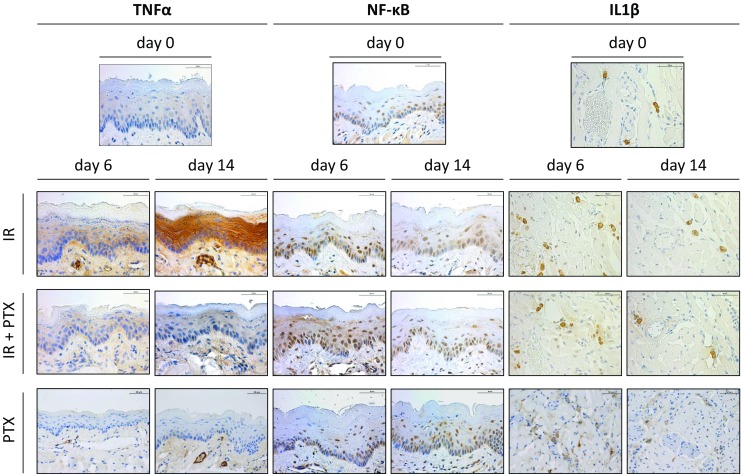
TNFα , NF-κB p50, and IL-1β expression during fractionated irradiation ± PTX and PTX alone. Representative histophotographs of lower mouse tongue stained for TNFα, NF-κB p50 (epithelial staining), and IL-1β-positive macrophages (deeper tongue tissue, in close proximity to blood vessels). Figures represent day 0 (unirradiated and untreated controls), day 6 and day 14 during fractionated irradiation alone (*IR*), with additional PTX administration (*IR+PTX*), and PTX treatment alone (*PTX*). PTX treatment reduced the radiation-induced expression increase of TNFα and IL-1β, but had no effect on NF-κB p50. Scale bar: 50 µm

Representative histophotographs of immunohistochemical staining for TNFα, NF-κB p50, and IL-1β in control specimen and on days 6 and 14 are presented in Fig. 1.

### TNFα

**Fig. 2:**
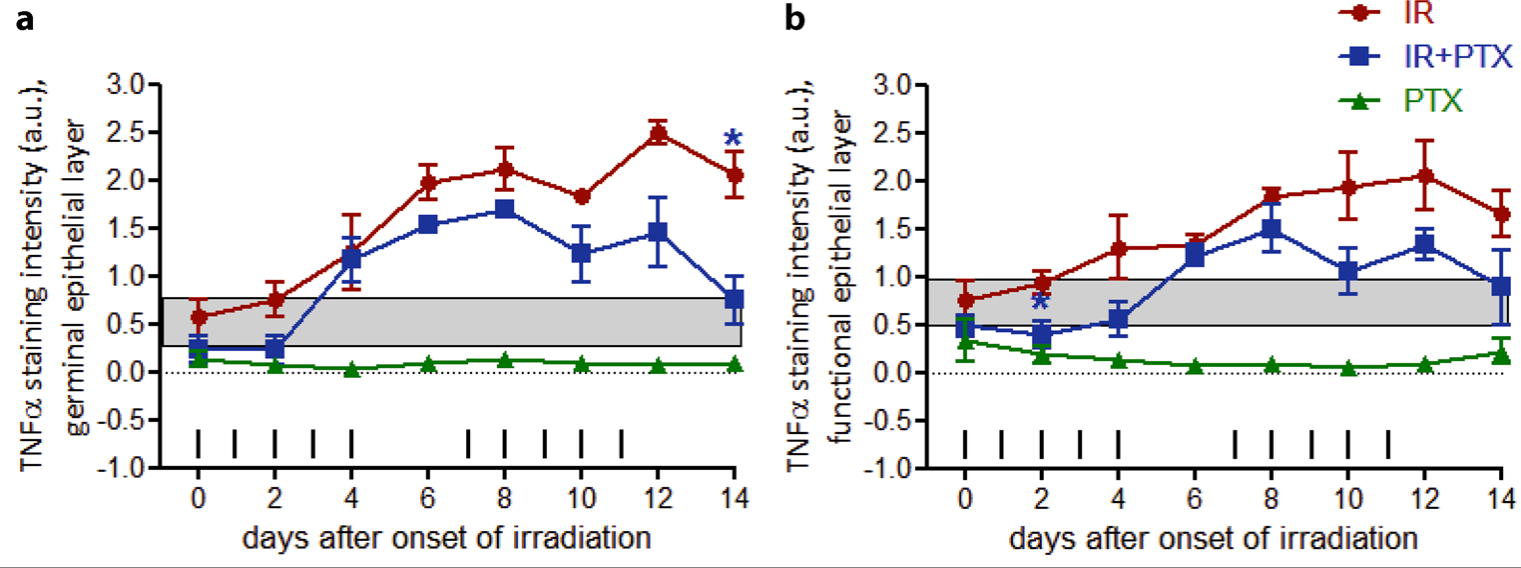
Effect of fractionated irradiation ± PTX and PTX alone on TNFα expression. TNFα expression was analyzed in the epithelium, in the germinal (**a**) and functional (**b**) compartments, respectively. The staining signal intensity was scored semiquantitatively with an arbitrary score of 0 (no signal), 1 (weak), 2 (intermediate), or a maximum of 3 (strong). Data points represent the mean of 3 animals, error bars indicate ±1 standard deviation (*SD*). The shaded areas illustrate the mean (±1 SD) from 3 control animals. The fractionation protocol is indicated on top of the abscissae. *PTX* pentoxifylline,* IR* irradiation. *p < 0.05

TNFα expression was minor in unirradiated and untreated control samples, with an average staining intensity of 0.6 a.u. in the germinal layer and of 0.8 a.u. in the functional compartment, respectively. With the onset of daily fractionated irradiation, as illustrated in Fig. 2, TNFα expression increased progressively. The expression maximum was observed on day 12 (2.5 a.u.).

In the germinal epithelial layers, additional PTX treatment reduced TNFα on day 0 (0.2 a.u.) and day 2 (0.2 a.u.) relative to only irradiated specimen (0.6 a.u. and 0.8 a.u.), respectively. Subsequently, the germinal expression in the PTX-treated experimental arm progressed similarly to only irradiated specimen until day 10, although the TNFα expression remained substantially lower compared to irradiation alone. The expression maximum on day 12 was reduced to 1.5 a.u. and on day 14, expression levels were found to be significantly below those for irradiation alone (*p* = 0.037) and within the normal range (0.75 a.u.) (Fig. 2a). In the functional epithelial compartment daily fractionated irradiation induced a progressive increase in TNFα expression with a maximum on day 12 (2.1 a.u.). With additional PTX treatment, TNFα expression developed similar to that after irradiation alone, but was constantly substantially lower, however, with a significant difference only being obtained at day 2 (*p* = 0.022) (Fig. 2b). PTX treatment alone reduced TNFα levels to subnormal values in both the germinal and the functional epithelial layers. Reduced epithelial TNFα expression remained throughout the PTX treatment time (Fig. 2a,b).

### NF-κB p50

**Fig. 3:**
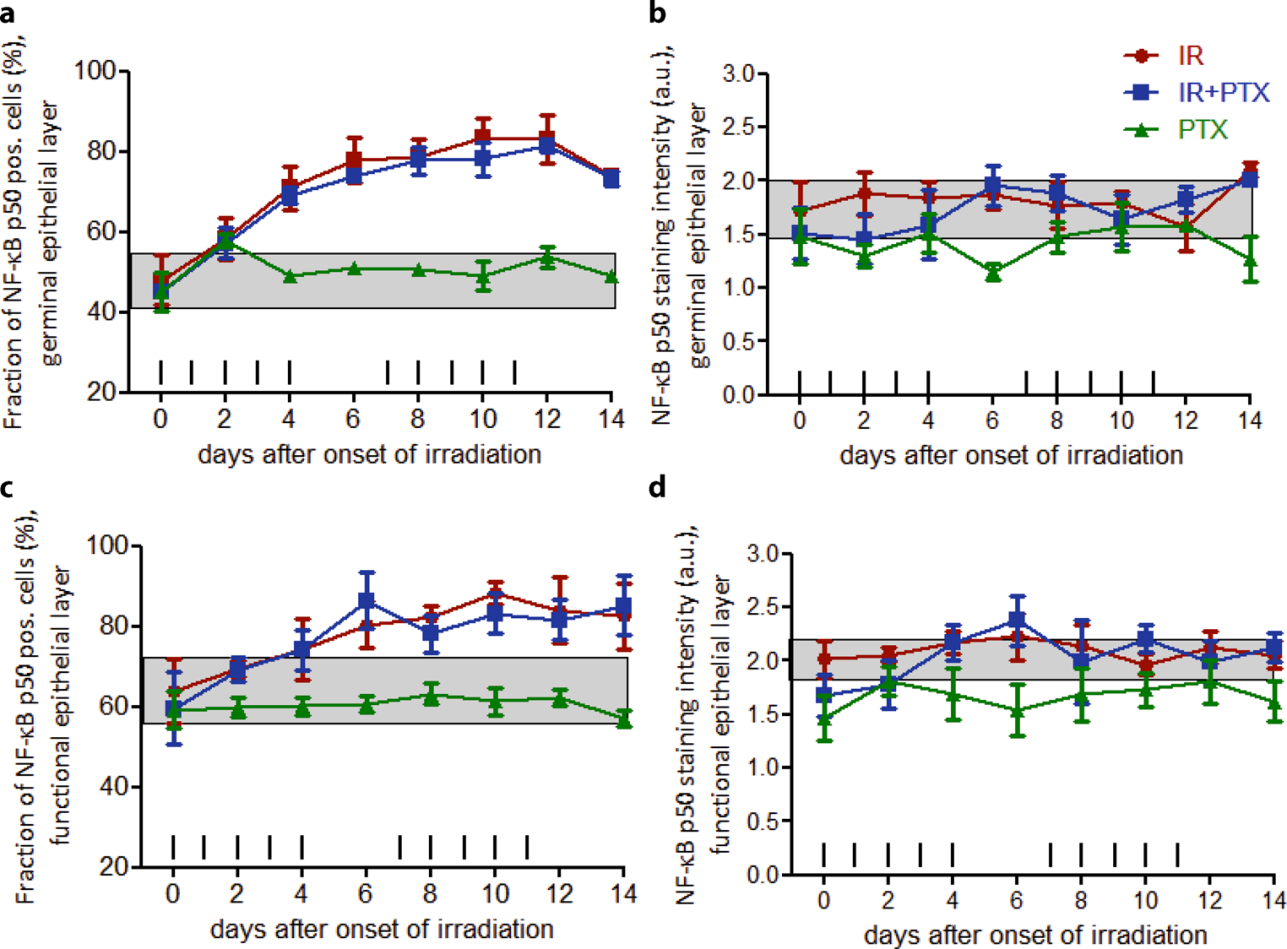
Effect of fractionated irradiation ± PTX and PTX alone on NF-κB p50 expression. NF-κB p50 expression was analyzed in the epithelium, in the germinal (**a**) and functional (**c**) compartments, respectively. The staining signal intensity was scored semiquantitatively with an arbitrary score of 0 (no signal), 1 (weak), 2 (intermediate), or a maximum of 3 (strong) in both compartments as well (**b** and **d**). Data points represent the mean of 3 animals, error bars indicate ±1 standard deviation (*SD*). The shaded areas illustrate the mean (±1 SD) from 3 control animals. The fractionation protocol is indicated on top of the abscissae. *PTX* pentoxifylline, *IR* irradiation

In control specimen, 47.9% of cell nuclei in the germinal epithelial compartment were NF-κB p50 positive. With daily fractionated irradiation, NF-κB p50 expression increased over the normal range on day 2 (58.3%) and progressively expanded to a maximum of 83% on day 12. NF-κB p50 staining intensity in the individual, positive cell nuclei was not affected by daily fractionated irradiation throughout the study period. Both the fraction of NF-κB p50 positive cell nuclei and their respective staining intensity remained unchanged by PTX treatment (Fig. 3a,b).

In the postmitotic, functional epithelial compartment, 67.2% of cell nuclei expressed NF-κB p50 in control sections. Daily fractionated irradiation progressively increased the NF-κB p50 expression to a maximum of 88.3% nuclei on day 10. NF-κB p50 staining intensity remained within the control range during the study period. Additional PTX treatment had no effect on the fraction of cell nuclei positively stained for NF-κB p50 or the respective staining intensity (Fig. 3c,d).

PTX treatment alone did not substantially affect the percentage of NF-κB p50 positive cell nuclei or their respective staining intensity. A minor reduction of the staining intensity in only the functional layer was observed (Fig. 3a–d).

### IL-1β

**Fig. 4:**
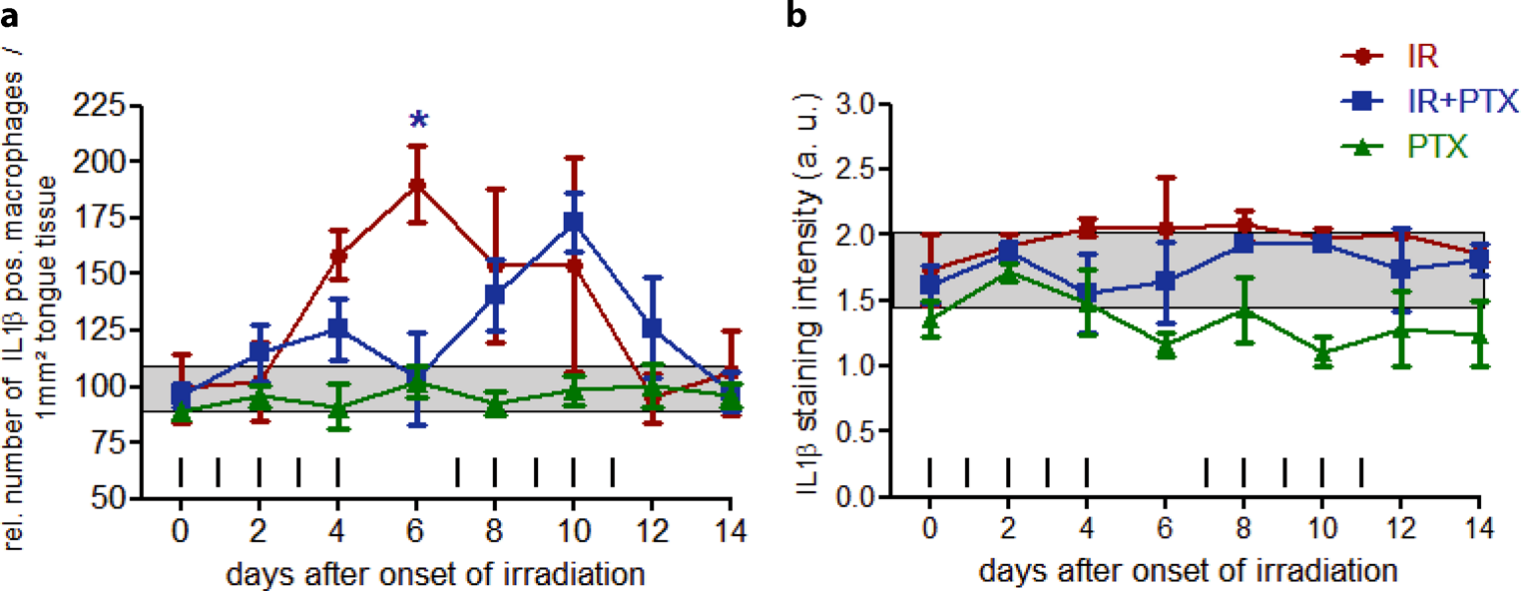
Effect of fractionated irradiation ± PTX and PTX alone on IL-1β expression in macrophages. IL-1β expression was exclusively found in macrophages (deeper tongue tissue). The number of macrophages positive for IL-1β (**a**) as well as the staining signal intensity (**b**) was evaluated. The staining intensity was scored semiquantitatively with an arbitrary score of 0 (no signal), 1 (weak), 2 (intermediate), or a maximum of 3 (strong). Data points represent the mean of 3 animals, error bars indicate ±1 standard deviation (*SD*). The shaded areas illustrate the mean (±1 SD) from 3 control animals. The fractionation protocol is indicated on top of the abscissae. *PTX* pentoxifylline, *IR* irradiation. *p < 0.05

Daily fractionated irradiation progressively increased the number of IL-1β positive macrophages to a maximum of 190% on day 6. Afterwards, the number of IL-1β positive macrophages declined despite ongoing irradiation, and normal values were restored on day 12 (Fig. 4a). IL-1β staining intensity did not change systematically throughout the study period. Initial values of 1.8 a.u. increased to a maximum of 2.1 a.u. on days 4–8. Subsequently, the staining intensity reentered the normal range.

Additional PTX treatment kept the number of IL-1β expressing macrophages close to or within the normal range until day 8. The IL-1β expression maximum was delayed to day 10 and reduced to 173%, relative to controls. However, the only significant difference to only irradiated specimen was found on day 6 (*p* = 0.022). The subsequent restoration of normal values was complete on day 14. IL-1β staining intensity remained lower, compared to only irradiated samples and within control ranges throughout the study period (Fig. 4b). PTX treatment alone had no significant effects on either the number of IL-1β positive macrophages or their respective staining intensity. Both parameters remained largely within the control range (Fig. 4a,b).

## Discussion

Virtually all head-and-neck cancer patients experience some degree of oral mucositis during their radio(chemo)therapy, the majority develops a severe, confluent reaction [1, 20]. Oral mucositis is associated with significant reduction of the patients quality of life, bears the risk for systemic infections, due to the loss of the epithelial barrier function, and for consequential late effects in the oral cavity [21, 22]. No biology-based treatment has so far been implemented into clinical practice.

PTX treatment during daily fractionated irradiation resulted in a significant reduction of the incidence of oral mucositis in the mouse tongue model [11] This may be attributed to a modulation of inflammatory processes which are suggested to be a major component of (early) normal tissue radiation effects [23, 24], including mucositis [25, 26]. Early inflammation is regularly observed during the clinical manifestation of oral epithelial ulceration in patients. NF-κB seems to play a key role in this aspect [8, 12]. With TNFα and IL-1β, stimuli for both the classical and the alternative NF-κB activation pathway have been investigated this study. Furthermore, both cytokines are involved in nociception [27]. PTX was found to ameliorate pain in preclinical as well as clinical studies, likely due to reduced TNFα and IL-1 release [28, 29]. This indicates that PTX might have an antihyperalgesic effect, reducing the significant mucositis-associated pain burden. This effect, however, is not substantiated in the animal model used in the present study.

### Irradiation alone

In our mouse tongue model, daily fractionated irradiation rapidly induced the expression of all inflammatory markers investigated. In contrast to TNFα and NF-κB expression levels, which remained considerably high throughout the study period, IL-1β was found to be downregulated at the end of the first treatment week, despite ongoing irradiation. This corresponds to the time of onset of repopulation, i. e., the regenerative response of oral epithelium to fractionated irradiation [10, 30]. A potential interaction with the epithelial regenerative radiation response is hence highly likely.

### Irradiation with additional PTX administration

PTX exhibits potent anti-inflammatory effects [31, 32], directly inhibits TNFα, and reduces inflammation in multiple preclinical models [33–35], chemotherapy-induced intestinal (CPT-11) and oral (5-FU) mucositis [36, 37]. In combination with daily fractionated irradiation, PTX had significant effects on TNFα and IL-1β, but no effect on the mutual downstream protein NF-κB. Being a central mediator of inflammation and other biological processes, NF-κB is regulated by multiple pathways [38]. TNFα and IL-1 are considered major NF-κB stimuli. However, TNFα is only one of the ligands of the tumor necrosis factor receptor (TNFR) superfamily, which consists of 19 ligands and 30 receptors [39], all of which result in the activation of the NF-κB pathway. In addition to the TNFR superfamily, Toll-like receptor (TLR) signaling can also result in NF-κB activation. TLRs react upon stimulation by damage-associated molecular pattern molecules (DAMP) and pathogen-associated molecular pattern molecules (PAMP) [40]. Especially DAMPs are likely to be released after irradiation and also PAMP signaling is likely due to changes in the host oral microbiome [41]. Hence, it appears to be highly likely that lacking TNFα and/or IL-1β is substituted by other stimuli. Activation could also occur via the atypical activation pathway. In addition to ligand-mediated activation, NF-κB can be stimulated by reactive oxygen species, such as hydrogen peroxide, which is abundantly produced during the radiolysis of intracellular water [42]. Further oxidants, such as singlet oxygen and superoxide and reactive nitrogen species have been shown to active NF-κB and are released by immune cells during inflammation [43]. This hypothesis is supported by the missing effect of specific TNFα inhibition with infliximab on oral mucositis, obtained in another study in the mouse tongue model [44].

The central role of NF-κB in the regulation of the inflammatory response promotes further targeting of this pathway as a treatment strategy to reduce radio(chemo)therapy-induced oral mucositis. Recently, a reduction of radiation-induced oral mucositis by specific targeting of NF-κB was demonstrated in the mouse tongue model [45].

### PTX alone

In our study, PTX treatment alone left the expression levels of all inflammatory mediatory investigated largely unchanged and within control ranges. PTX most likely exerts its mucositis-ameliorating activity through a mechanism other than modulation of NF-κB associated inflammation.

Presumably, the recently demonstrated PTX-mediated reduction of radiation-induced early epithelial hypoxia [19] accounts for the beneficial effect. Further mechanisms, e. g., modulation of cell junctions or increased epithelial proliferation could also contribute. Modulation of epithelial proliferation by PTX is currently being investigated.

## Conclusion

Based on these results, the mucositis-ameliorating effects of PTX, observed in functional studies [11], cannot be attributed to a reduction of radiation-induced NF-κB associated inflammatory changes. Nevertheless, reduced TNFα and IL-1β expression could alleviate mucositis-associated pain sensation. The anti-inflammatory approach remains a promising treatment strategy for the oral mucositis. Further analyses of the mechanistic effects of PTX during the development of radiation-induced oral mucositis are required to fully elucidate its potential as mucositis-ameliorating treatment strategy.
